# Defining Internal Tissue Closure: High-Resolution Ultrasound Evaluation of Interi—A Novel Internal Tissue Closure System

**DOI:** 10.1093/asjof/ojac073

**Published:** 2022-09-20

**Authors:** David Alfonso, Bradley Bengtson, Patricia McGuire

**Affiliations:** plastic surgeons in private practice, Grand Rapids, MI, USA; plastic surgeons in private practice, Grand Rapids, MI, USA; Plastic surgeon in private practice, St. Louis, MO, USA

## Abstract

**Background:**

Seroma remains a leading postsurgical complication in plastic surgery. Conventional drains are ineffective in clearing blood and fluid and closing down surgical spaces. The Interi (Internal Closure System, IC Surgical, Grand Rapids, MI) is comprised of a novel branching internal manifold attached to a self-contained portable pump with a higher, consistent, continuous negative pressure, may reduce this long-standing issue. In addition, high-resolution ultrasound (HRUS) has emerged as an ideal tool to visualize structures, fluid collections, and seromas internally.

**Objectives:**

This study evaluates Interi in full abdominoplasty patients utilizing HRUS to evaluate Interi's ability to evacuate blood and fluid, hold internal tissues together and document, for the first time, what internal tissue healing actually looks like radiographically.

**Methods:**

An IRB approved, Contract Research Organization reviewed retrospective study evaluated consecutive patients undergoing full abdominoplasty utilizing Interi from July 2020 through March 2021 by three plastic surgeons. HRUS visualized and confirmed the presence or absence of fluid collections and healing tissue planes during the postoperative process. Study data and all adverse events were recorded, with HRUS images reviewed by investigators and confirmed by an independent radiologist.

**Results:**

Seventy-one Interi patients were enrolled. Mean age was 43 (range: 21-74) and BMI was 28. Seroma was confirmed clinically and through HRUS in 3/71 patients, and was associated with either clot (2) or failure to activate system (1). Interi's ability to eliminate fluid and approximate/hold surgical tissue planes together was confirmed with HRUS. No other major complications, including abscess, hematomas, or flap necrosis were observed.

**Conclusions:**

This novel Internal Tissue Closure System effectively evacuated blood and fluid, approximated and maintained closure of internal tissue planes in abdominoplasty patients, allowing for primary tissue healing and internal wound closure to occur. Healing tissue planes and any fluid present are easily identified on HRUS visualizing actual internal tissue healing with a simple, widely available radiographic scan.

**Level of Evidence: 3:**

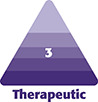

Failure to achieve approximation and internal closure of tissue planes and deep surgical spaces where anatomical dead space is created can lead to both short- and long-term wound healing adverse events. Early fluid collection within these tissue planes and eventual seroma formation is a leading complication of surgical procedures when internal tissue planes are not effectively evacuated, approximated, and closed. These fluid collections and seromas often cause increased patient pain and morbidity, require multiple percutaneous aspirations, additional device placement, and added surgical procedures. Conventional drains have been used by surgeons for the past 75 years,^1,2^ but persistently high reported rates of seroma and poor wound healing continue and are indicative of their ineffectiveness in closing down and healing of surgical spaces.^3,4^ Current devices access only limited areas of the surgical space, and generated negative pressure delivered from current drains is inconsistent and inadequately low to hold together, and actively facilitate closure of postsurgical tissues.^5,6^ These limitations often result in residual blood and fluid collection within the operated area, lack of tissue approximation, eventual seroma formation, and other postoperative adverse events that result in poor tissue healing and potential long-term adverse events.^7–10^

We utilized a novel system, Interi (IC Surgical, Grand Rapids, MI), that includes an extruded silicone manifold with a central trunk along with 3 additional “peel-apart” channel creating 4 distinct branches connected to a therapy unit to deliver a continuous −125 mmHg negative pressure to internal tissue planes while simultaneously removing excess fluid from the surgical spaces (Figure 1). By effectively removing blood and fluid at a higher negative pressure, the tissue planes are drawn in, approximated, and held together, allowing for primary tissue healing to occur unhindered without detrimental fluid collection or seroma formation.^5,11^

**Figure 1.**
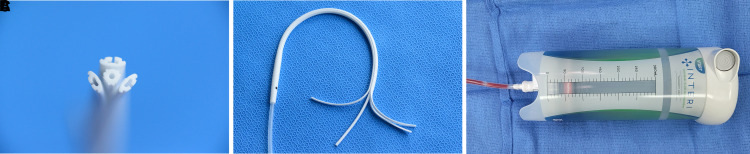
The Interi system (Internal Closure System, IC Surgical, Grand Rapids, MI) consists of a manifold (A, B) with 4 branches that connects to a single-use therapy unit (C) which is a closed, disposable device maintaining a constant −125 mm HG pressure. (A) Tip of manifold branches showing channel design; (B) branches partially peeled apart; (C) 300 mL capacity Interi therapy unit is depicted. Current sizes include 150, 300, and 500 mL capacity.

High-resolution ultrasound (HRUS) has been utilized by plastic surgeons and radiologists since the 1980s to diagnose, locate, evaluate, and treat active postsurgical fluid collections and seromas. Most recently, HRUS is gaining momentum in plastic surgery for the diagnosis of breast implant shell failure, fat transfer, cellulite evaluation, and importantly, diagnosis and treatment of fluid collections. HRUS is becoming, at a minimum, an equivalent dynamic alternative to MRI or computed tomography for these assessments vs standard clinical examination or more expensive static radiographic tests.^12–16^ HRUS was utilized in this study in all subjects as part of the investigators' standard of care postoperatively (plastic surgery imaging, 12 MHz transducer; Figure 2).

**Figure 2.**
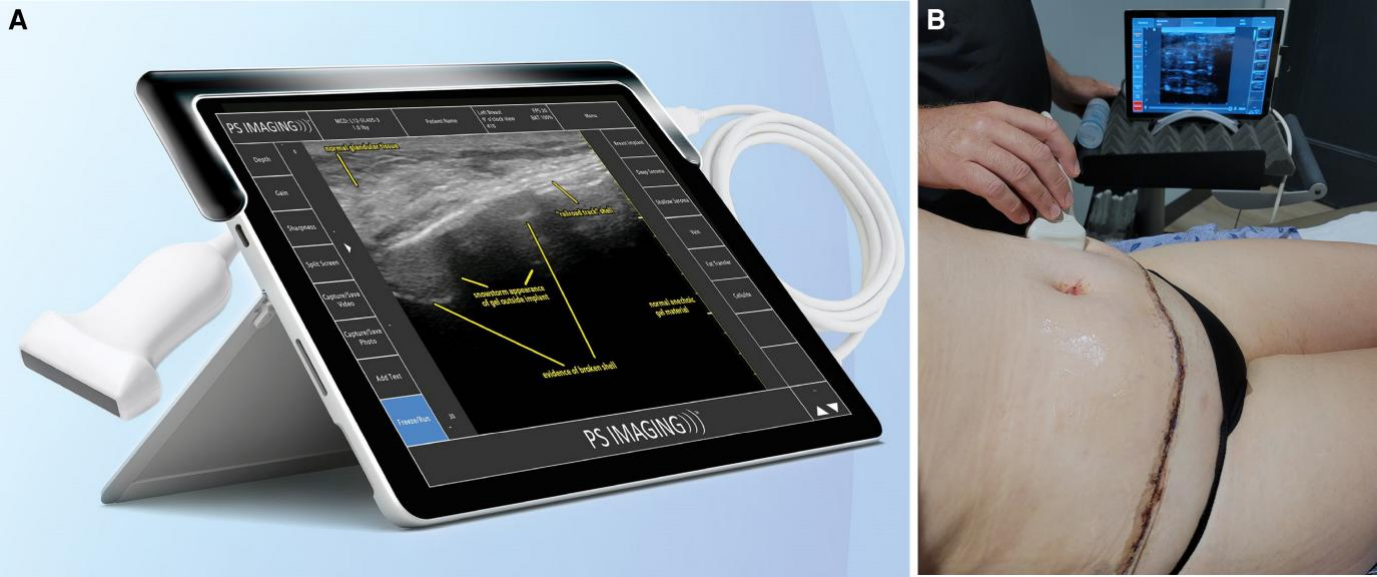
(A) Plastic surgery imaging high-resolution ultrasound imaging system utilized for the study with 12 MHz transducer. (B) Scanning a 36-year-old female patient's abdomen postoperatively on the day of Interi (Internal Closure System, IC Surgical, Grand Rapids, MI) removal.

The objectives of this consecutive retrospective case series are to: (1) evaluate the ability of Interi to fully evacuate postoperative effluent and blood from within the surgical space documented with HRUS; (2) evaluate the ability of Interi to approximate and hold together surgical tissue planes in a series of abdominoplasty patients confirmed on ultrasound; (3) document adverse events and complications in the series including fluid collection and seroma compared to historical outcome studies; and (4) document, demonstrate, and define what internal tissue closure actually looks like radiographically utilizing HRUS.

## METHODS

IRB approval (WCG IRB #20213835) was obtained for a retrospective review of the first 71 consecutive full abdominoplasty patients utilizing the Interi system from July 2020 through April 2021, by 3 board certified plastic surgeons in 2 separate surgical practice locations. The board found that this research met the requirements for a waiver of consent under 45 CFR 46 116(f)[2018 Requirements] 45 CFR 46.116(d). Study population was defined as all patients 18 or older who underwent full abdominoplasty with utilization of the Interi^R^ system from July 1, 2020 through April 30, 2021 under the care of the three investigators at two sites. All patient data and full charts were confirmed and reviewed at each site, in person, by an independent Clinical Research Observer (M-Squared Associates Regulators, New York, NY). HRUS (Acertara Acoustic Laboratories, Longmont, CO—12 MHz Transducer) was used as routine practice by the three surgeons to visualize whether any fluid collections were present in the surgical site at the time of manifold removal and postoperatively with any signs of swelling or clinical suspicion of seroma. Along with thorough 4 quadrant scanning of the abdomen, any clinical swelling was evaluated with HRUS, and any visible fluid collections documented. For each patient, patient BMI, age, sex, prior abdominal surgery or abdominal liposuction, concurrent liposuction at the time of this procedure of either the flanks or the anterior abdomen, use of transversus abdominis plane (TAP) blocks, pain pumps, days to manifold removal, number of therapy units utilized, and total fluid collected was recorded. Along with fluid collections or seroma, any minor or major wound healing complications were also recorded including suture abscess, cellulitis, deep infection, bleeding or hematoma, skin or flap necrosis, or need for any early surgical revision. All adverse events were recorded. Specific evaluation and visualization of the healing tissue planes with HRUS was performed. HRUS images were recorded in the medical records for 24 patients. Recorded HRUS images were also reviewed by an independent Board Certified Radiologist without any conflicts of interest.

**Figure 3.**
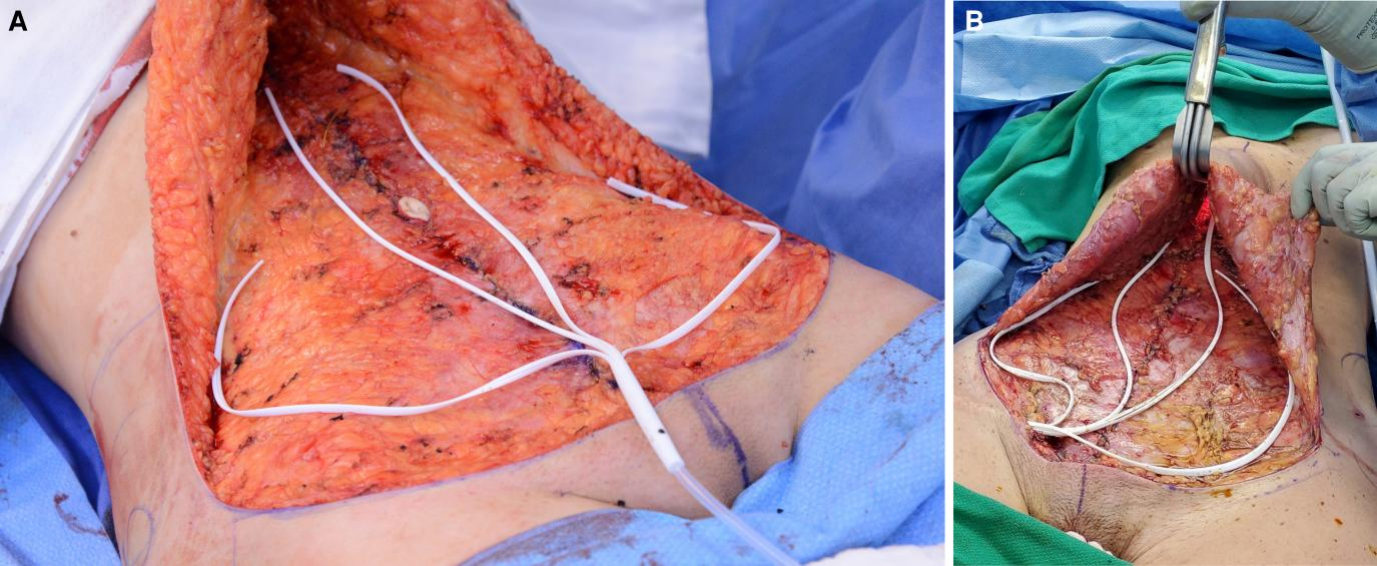
The internal manifold is in position in a 31-year-old female patient, allowing broad distribution of −125 mm HG negative pressure throughout the entire surgical space bringing the manifold out (A) in the suprapubic position and (B) just above the right-hip flexion crease in a 42-year-old female.

**Figure 4.**
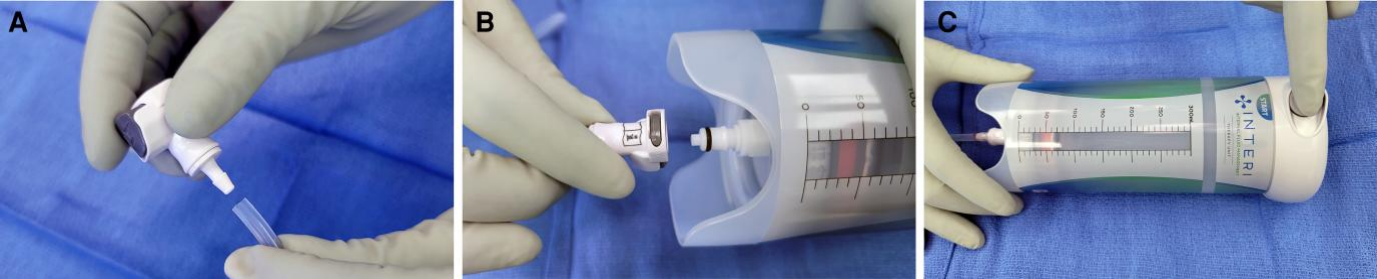
(A) Following incision closure the manifold tubing is attached to the connector. (B) The connector is then attached to the Interi therapy unit (Internal Closure System, IC Surgical, Grand Rapids, MI) seating in with a “click.” (C) The therapy unit has a loaded spring that upon activation maintains the internal pressure at a constant −125 mmHG.

Full abdominoplasty with elevation of the abdominal flap to the xiphoid was performed by all 3 surgeons with Bovie Cautery. Ultrasound-guided TAP blocks placed by the anesthesia team along with use of pain pumps were recorded. The Interi system has a branching manifold with 4 branches that are peeled apart and placed throughout the surgical site. Each manifold was completely opened with all 4 branches placed throughout the entire surgical space to maximize full coverage: one manifold branch along each side of lower abdomen extending into each gutter and the other two branches running vertically 2 to 3 cm off the midline (Figure 3). No branches were trimmed. No progressive tension sutures (PTSs) or sutures to secure the manifold branches were placed. Tubing was tunneled from beneath Scarpa's fascia a minimum of 6 cm obliquely up through the skin using the preattached trocar, and the exit site created in the suprapubic area or above the right-hip flexion crease. Manifold tubing was placed to full wall or full Neptune suction (Stryker, Kalamazoo, MI) until incision closure was completed and dressings applied, at which time the therapy unit was attached through the connector and activated just prior to leaving the operating room. At the completion of the procedure, the sterile connector was attached to the tubing and the connector then attached to the therapy unit and activated (Figure 4). The patients were educated on recording fluid volume, monitoring, and exchanging the therapy units at the time of their initial consult, immediately preoperatively and postoperatively, and video instructions supplied. Therapy units were changed when full and the manifolds removed when output volumes decreased to 20 cc or less for the prior 24 hours.

## RESULTS

A total of 71 consecutive full abdominoplasty patients receiving the Interi system (Internal Closure System, IC Surgical) from three plastic surgeons were studied. There were 68 females (95.8%) and 3 males. The patient age range was from 21 to 74 years, with an average age of 42.3 years. The BMI ranged from 18.7 to 38.4 with a mean of 27.6. Four patients (5.6%) had prior liposuction of the abdomen and 6 had a prior abdominal surgery (8.5%). Fifty-nine of 71 patients (83.1%) had concurrent liposuction of the flanks primarily performed through a separate lateral incision with average of 509 mL of tumescent fluid used, and 22 of 71 (31%) had liposuction of their anterior abdomen with average of 1054 mL of tumescent fluid used (Table 1).

**Table 1.** Patient Demographics—Past and Concurrent Procedures

Patient Demographics	Number (%)
Surgeons	3
Patients	71
Female	68 (96%)
Male	3 (4%)
Age	
Mean	42.3
Range	21-74
BMI, kg/m^2^	
Mean	27.6
Range	19-44
Prior abdominal procedures	10 (14%)
Prior liposuction of the abdomen	4 (4%)
Prior abdominoplasty	6 (7%)
Transversus abdominis plane	53 (75%)
Pain pumps	0 (0%)
Concurrent liposuction of the flank	59 (83%)
Concurrent liposuction of the anterior abdomen	22 (31%)

Ultrasound-guided TAP blocks were performed for postoperative pain control by the anesthesia team in 53 patients (75%) from 2 surgeons. No pain pumps were utilized in any patients. The exit site was placed in the suprapubic area in 49 patients (69%) and the right lateral incision area above the hip flexion crease in 22 patients (31%; Table 1).

The length of therapy on Interi, calculated as the number of days from surgery until manifold removal, ranged from 5 to 18 days with an average of 9 days. In our prior 100 standard drain patients, the average drain time was 12 days. Total fluid output averaged 570 cc with a range of 90 to 1550 cc. Additional therapy units are included in each kit to cover each patient's postoperative course. Average postoperative follow up was 6.3 months, ranging from 1.8 to 10.2 months, July 2020 through May 2021 (Table 2).

**Table 2.** Interi Duration, Volumes Collected, and Study Period

Variable	Statistics
Duration of therapy	
Mean (days)	9.1
Range (days)	5-18
Fluid collected	
Mean (mL)	570
Range (mL)	90-1550
Postoperative follow up	
Mean (months)	6.3
Range (months, July 2020-May 2021)	1.8-10.2

**Figure 5.**
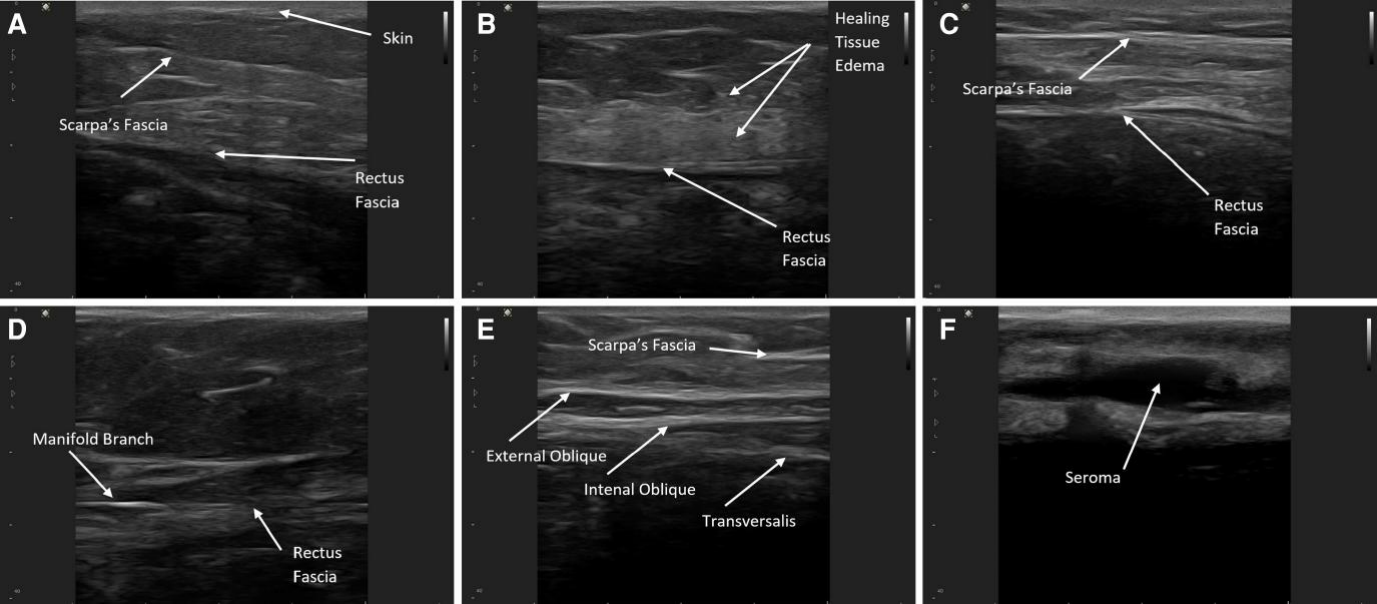
(A-E) Sample high-resolution ultrasound (HRUS) images (Plastic Surgery Imaging—12 MHz transducer) from the first 71 consecutive Interi system (Internal Closure System, IC Surgical, Grand Rapids, MI) patients recorded at the time of device removal showing no fluid collection with documented anatomy. Skin, Scarpa's fascia, and abdominal fascia are marked. (F) One of the 3 postoperative patients that developed a seroma. She failed to activate the new therapy unit on postoperative Day 3.

**Figure 6.**
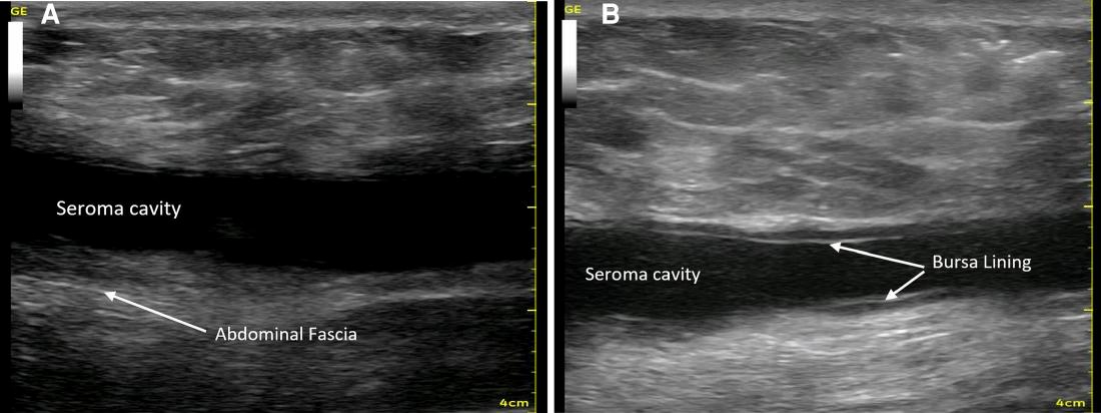
(A) Typical seroma appearance on high-resolution ultrasound (HRUS), prior to the use of Interi (Internal Closure System, IC Surgical, Grand Rapids, MI), showing ∼200 cc of fluid 3 weeks following an abdominoplasty with prior standard of care Blake drain. (B) Seroma cavity after partial drainage with evidence of a pseudocyst lining. Video 2 shows ultrasound-guided drainage.

HRUS was performed routinely by all three surgeons on the majority of patients either on the day of manifold removal or subsequent visits. In the first 5 patients, additional ultrasounds were performed throughout the first week secondary to the systems first use clinically. Sixty-six of 71 patients had HRUS documented and images from 24 patients were recorded and saved in the patient's chart. On subsequent postoperative visits, HRUS was performed in patients with any swelling or clinical suspicion of seroma. In our first 71 patients, 3 patients had clinical seromas following device removal (4.2%; Table 3). Two patients had a clot in the tubing that was not detected and resulted in clogging of the tubing, and 1 patient failed to push the start button to initiate the therapy unit after exchanging the unit, resulting in fluid collection. Seromas were detected and treated on postoperative Days 14, 18, and 23 with 1 to 3 aspirations required. These issues did not recur with improved patient education and improved device surveillance. Twenty-four patients' HRUS images were recorded and sample patient ultrasounds are depicted (Figure 5A-E). Recorded HRUS obtained throughout the postoperative course were evaluated by an independent radiologist with no fluid identified except in the 3 patients in whom surgeons had also noted fluid collections. Figure 5F shows a seroma in one of the 3 patients in our study who had an ultrasound after failing to restart the system after changing her therapy unit. Figure 6 depicts a chronic seroma in one of our immediate prior 100 standard drain patients depicting seroma fluid plus a well-developed pseudocyst lining. Attached videos demonstrate ultrasound drainage of this seroma.

**Table 3.** Seromas

Seroma-related complications	No. of patients (%)
No seroma or fluid collection	68 (96%)
Seroma fluid visible on high-resolution ultrasound	3 (4.2%)
Long-term >30-day seroma following treatment	0 (0%)

No patients had any clinical seroma or visualized fluid on HRUS after 30 days postoperatively, including the 3 patients with seromas following treatment. Reviewing 100 standard drain patients immediately preceding this ICS study, 22 patients had seromas that were treated with aspiration. Two videos of one of the original seroma patients being drained by ultrasound guidance are included (Videos 1, 2).

Patient charts were reviewed for all postoperative complications including: suture abscess, cellulitis, deep infection, hematoma/bleeding, skin or flap necrosis, and need for early surgical intervention and other adverse events. There were 10 patients with complications (14.1%). No patients had any major complications that required further surgical revision during the study period. One of the 3 seroma patients had associated cellulitis that cleared with oral antibiotics. Two additional patients had cellulitis clearing with one course of oral antibiotics. Two patients had small umbilical dehiscence healing without additional surgery. One patient had prolonged upper abdominal muscle spasm without fluid or other identifiable etiology that resolved spontaneously. One patient had a superficial sterile suture abscess that resolved after suture removal. No other complications were noted in any of the 71 patients (Table 4). Also of note, no patients suffered any skin flap necrosis or evidence of compromised perfusion.

**Table 4.** Other Wound Complications

Complication	No. of patients (%)
Suture abscess—healed with suture removal	1 (1.4%)
Cellulitis—cleared with 1 course of antibiotics	3 (4.2%)
Umbilical dehiscence	2 (2.8%)
Muscle pain epigastric	1 (1.4%)
Deep infection	0 (0%)
Bleeding/hematoma	0 (0%)
Skin flap necrosis	0 (0%)
Need for early surgical revision	0 (0%)

**Figure 7.**
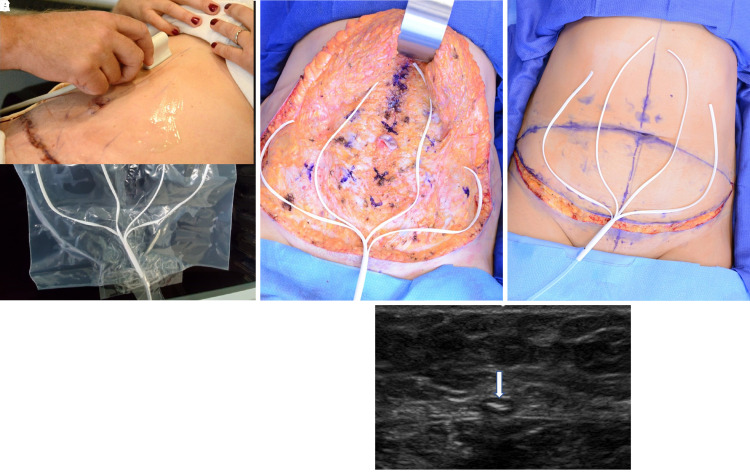
(A) As an example of manifold branch maintenance, fully opened manifold branches were placed inside a 5-gallon zip-lock bag and placed on Interi (Internal Closure System, IC Surgical, Grand Rapids, MI) suction. The branches remained in position while on suction. (B and C) Markings of manifold position were transposed to the overlying skin and followed for position maintenance postoperatively in this 32-year-old female. (D) High-resolution ultrasound (HRUS) confirming manifold position in its original location until device removal as shown in this 36-year-old female. (E) Cross-section HRUS image of a manifold laying on the abdominal fascia with no associated surrounding fluid.

In addition to ruling out fluid collections, ultrasound may also be as a tool for detection and evaluating manifold position during therapy. Early in the series, the first 5 patients' manifold positions were identified postoperatively after transposing and marking their position onto the anterior skin surface intraoperatively. Ultrasound confirmed the internal manifold branches remained in their surgically placed positions without the need for intraoperative suturing postoperatively, and may easily be identified including on cross section with no visible fluid surrounding the manifold branches until device removal (Figure 7).

## DISCUSSION

Postoperative fluid collections and subsequent seroma formation leading to poor wound healing continues to be one of the most significant complications reported in nearly every surgical study published today. There is even more at risk in patients undergoing extensive surgical procedures, patients with Acellular Dermal Matrices in the abdominal wall or breast, orthopedic implants, and specifically patients undergoing prepectoral breast reconstruction in which seroma can significantly affect recovery and morbidity.^17–22^

Clinical evaluation alone to address internal fluid collections and seroma may not be reliable. Surgical drains have significant limitations: they have access to only one specific space adjacent to the single-arm tubing; they do not generate sufficient or consistent pressure to draw and hold tissue planes together; and thus are largely unsuccessful at closing down surgical spaces and eliminating subsequent seroma formation. Two large multivariate analysis papers studying seromas with prior seroma reduction methods including Scarpa's fascia preservation, surgical glues, and PTSs have been published.^17–30^ The first by Ardehali and Fiorentino did a multivariate analysis of 15 separate studies including 1824 total patients.^24^ Only 5 of 15 studies used any form of HRUS, the other 10 used clinical examination only. Scarpa's preservation studies showed no statistical differences, PTS studies showed a reduction of seroma from 21.5% without PTS reducing to 6.2% (15/260 patients) with PTS. Tissue glues were able to reduce seroma only to 11.1% (16/140 patients) from 21% (25/121 patients) in patients without glue.^24^ Further, in their review of 15 these studies and the overall literature, they found seroma rates ranging from 1% to 57% and stated a generally accepted rate in the abdomen with standard drains without specific seroma-reducing methods are reported to be approximately 10%.^23–30^ Surgeons who frequently perform body contouring procedures know their seroma and general complication rates. Our review of the literature and our pre-Interi practice experience, along with the majority of these multivariate studies have the average seroma rates following abdominoplasty in the 18% to 22% range with standard of care drains without any other seroma-reducing techniques.^1–12,16–19,23–39^ This is consistent with our pre-Interi seroma rate of 22% in the 100 consecutive patients directly prior to this study. Videos of standard drain seromas are included (Videos 1, 2). Clinically evident seromas are usually dramatic, greater than 100 cc, with the vast majority requiring sequential draining 2 to 3 times weekly, for 2 to 3 weeks or placement of a seroma catheter or another device (Figure 6). Cohera Tissuglu (Cohera Medical, Inc., Oplotnica, Slovenia) FDA Premarket Approval Study actually reported a higher seroma rate, 22%, in their standard of care group: 2 Blake drains plus Cohera Tissuglu, vs the standard of care: 2 Blake Drain only group of 18%.^40^

In abdominoplasty, an effective approach to reducing seroma has been PTS techniques. PTS have been reported to reduce or eliminate seroma in a number of studies and have many strong proponents. Very low seroma rates have been reported (0%-2%) in may large studies since Pollock and Pollock's original publication.^25–36^ Scarpa's fascia preserving techniques with limited lateral dissection along with PTS techniques has lowered seroma rates further. Additional benefits of PTS include those associated with a drain or ICS including exit-site irritation and the inconvenience of wearing a fanny pack. More recently, in procedures where tissues can easily be approximated such as the abdomen, it can be a very powerful tool. However, in thin patients, they may cause visible indentations, dimpling, and contour deformities of the skin, although the majority of these resolve. PTS also adds extra operating time (15-30 minutes) to the procedure with operating time ranging from a minimum $20USD per minute to over $400USD per minute at many hospital centers.^24–29^ In addition, not all surgical procedures are amenable to PTS such as breast reconstruction, and where large volumes of tumescent fluid and liposuction are performed, the fluid is not effectively evacuated. Disadvantages of the ICS system include added cost of the entire kit with 3 therapy units, currently ∼$700, discomfort and potential scarring at the exit site and the inconvenience of monitoring and changing the therapy unit along with carrying the fanny pack. There was no charge for HRUS and the follow up for ICS patients is the same or less than standard drain patients. There are always trade-offs with techniques in surgery and surgeons continue to weigh these advantages and disadvantages daily.

Our first prospective pilot study use of the ICS resulted in no seromas in a prospective study of 24 full abdominoplasty patients.^11^ In this current study, ICS utilization for full abdominoplasty in 71 patients by 3 surgeons, 3 patients (4.2%) developed a seroma. The 3 seromas were noted. Two patients had clogged tubing and 1 patient failed to start the new therapy unit after exchange. In all other patients, ICS effectively evacuated postoperative effluent and blood from the internal surgical site as evidenced by no fluid collections in 68 of 71 (96%) patients on clinical examination and confirmed with HRUS (Figure 5). The channeled manifold branches that transmit the negative pressure in the surgical space were observed to remain in their originally placed position and confirmed on HRUS in the first 5 patients of this study and the first 8 patients in our prior prospective pilot study. This is further demonstrated by placing the manifold branches in a large Ziploc (SC Johnson, Racine, WI) bag on suction delivered by the ICS without movement (Figure 6).

The benefits of negative pressure stimulation of tissue are well established in the literature with the vacuum-assisted closure system, and by delivering negative pressure directly to the internal surgical site, the Interi system has internalized this process and may offer similar clinical benefits to internal tissues.^37^ Importantly, this study also uses HRUS, for the first time, to visualize the actual healing tissue planes and document what internal tissue closure looks like radiographically (Figure 7).

Of additional interest, plastic surgery nurses and surgeons have noticed the overlying abdominal skin flaps have visibly reduced edema and are much softer and more pliable in patients who received ICS. This is difficult to quantify but may be the result of Interi clearing extracellular fluid in the overlying skin flaps. Following tissue approximation, after eliminating fluid and with no dead space, ICS is likely removing the extracellular fluid from the overlying anterior skin flaps, thereby reducing flap edema. Similar to Prevena (3M-KCI, San Antonio, TX) improving blood flow to surgical skin flaps by applying external negative pressure to the skin, the internal application of negative pressure may also have contributed to the zero incidence of skin flap necrosis in this study. No patients in our study suffered skin flap wound healing issues or required surgical revision.

The greater the tumescent fluid utilized, the greater the potential fluid development and output, as not all of the fluid within the tissues is absorbed or is passively eliminated without active removal. This is one of the major advantages of ICS. There are also emerging studies of increased seromas with ultrasonic liposuction and superficial body contouring.^39^ Some new user surgeons are using Interi for seroma prevention in these patients. It will be interesting to study whether prior abdominoplasty or liposuction procedures of the abdomen and/or higher BMI patients do in fact have higher outputs, and whether ICS can lower longer term adverse events in these subsets of challenging patients. A recent 2021 study by Vasilakis et al did show higher complications across the board in BMI patients greater than 30 kg/m^2^ including seroma rates of 18.5% in the higher BMI group vs those patients with a BMI of less than 30.^31^ However, the lower BMI patients still had a 16.6% seroma rate overall with standard drain technology. Their study showed in patients that required early operative intervention, over 38% were seroma-related surgical revisions. In our study, Interi did effectively clear even high volumes of postoperative fluid in patients, where larger volumes of tumescent fluid were used and liposuction performed, including 2 patients with total output of 1550 cc during the course of their treatment. In our prior prospective pilot study, 1 surgeon used pain pumps and 1 patient had 2.5 L of output and none of these original 24 patients had any seroma formation, with ICS clearing all of the fluid in the surgical space including this high volume patient.

HRUS is quickly becoming the standard of care for plastic surgeons looking for adverse events postoperatively verses relying on clinical examination only.^12–16,20,24,30^ There is no question that fluid collections can be identified much more accurately with HRUS vs standard clinical examination alone. Figure 6 depicts a seroma following abdominoplasty with prior drain standard of care, and its appearance on HRUS (Video 2). HRUS is quickly gaining popularity among plastic surgeons and should become the standard of care in breast and body contouring procedures and future research when reporting postoperative fluid and seroma complications, vs clinical examination alone.

**Figure 8.**
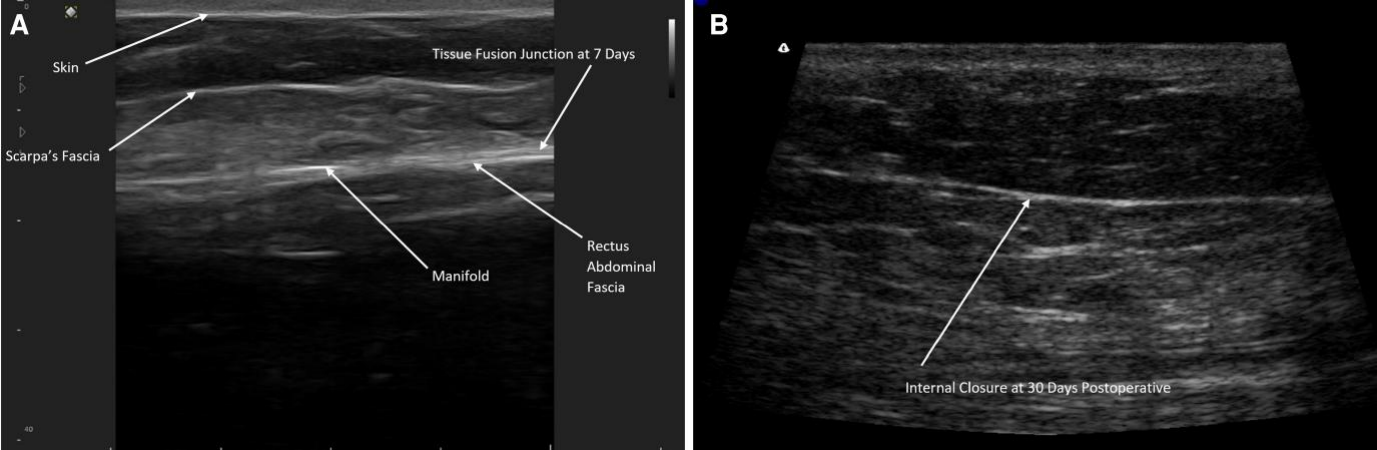
Formal documentation of the plane of tissue closure with overlying abdominal flap healing to the underlying fascia with no fluid collection present. Internal tissue closure on high-resolution ultrasound (HRUS) shown on (A) postoperative Day 7 and (B) postoperative Day 30.

HRUS easily identifies any and all fluid present, healing tissue planes, Scarpa's fascia, abdominal fascia, manifold branches, and most importantly the approximated deep tissue plane, where the overlying skin flap junction is healing to the abdominal fascia. We can now identify this plane or zone of tissue healing and show and accurately define what internal tissue closure looks like radiographically (Figure 8).

Recently published outcomes in immediate prepectoral breast reconstruction with ADM utilizing ICS vs standard drains has reduced seroma rates from 20.5% in drain patients to 0, in 23 Interi patients and 38 breasts.^41^ This study is being expanded to include 100 patients in each group. All studies have limitations and our study has no formal comparison group, although major complications were compared to our immediate standard drain patients similar to the breast reconstruction study by Paul. To address these concerns, future clinical studies may include comparing ICS vs PTS directly using HRUS and looking at costs, and all specific complications and cosmetic outcomes and duration of healing. Additional implant-based breast reconstruction with acellular dermal matrix and surgical scaffolds and other surgical applications including abdominal wall reconstruction, intra-abdominal oncologic and trauma surgery, and future replacement of chest tubes with ICS technology.

## CONCLUSIONS

**Figure 9.**
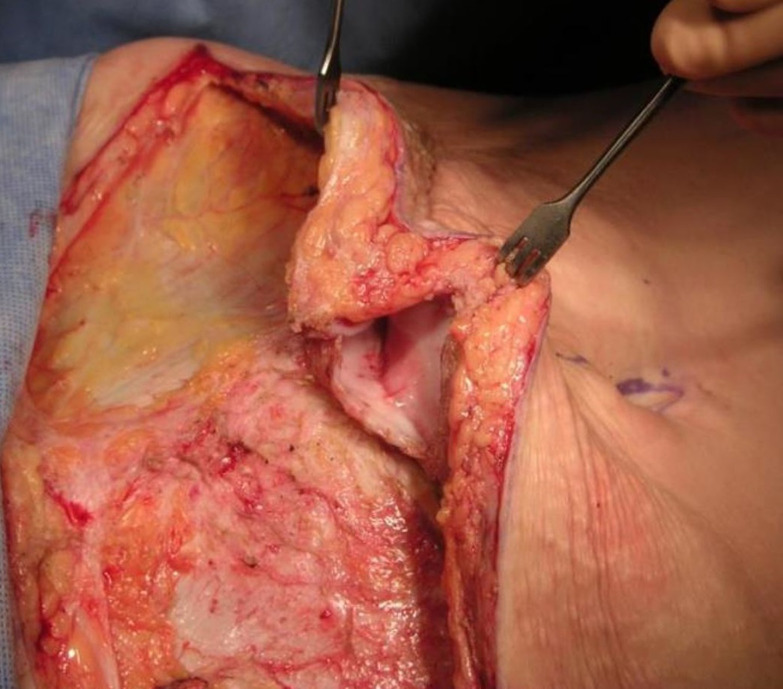
Seroma formation following standard drain placement may lead to a variety of postoperative complications. Old seroma fluid pockets are commonly seen in revisional surgery particularly in the abdomen and breast and a lined “pseudocyst” seroma is shown here at the time of revision abdominoplasty in this 45-year-old female.

Interi was developed to address long-standing issues of poor healing and postoperative complications associated with internal fluid collections. Current drain technology attempting to address this issue has failed to eliminate seroma with drain technology essentially unchanged for 75 years. Seroma cavities are commonly encountered in revisional surgery particularly in the abdomen and breast, indicative of prior poor healing stemming from seroma formation (Figure 9). Poor internal healing is the primary driver of why this technology was created. It confirms that by distributing a higher, consistent, and constant negative pressure broadly throughout the surgical space, blood and fluid may be more effectively evacuated, tissue planes closed and held in approximation by this ICS allowing for primary tissue healing and internal wound closure to occur.

The objectives of this consecutive retrospective case series were all met: (1) In our first 71 consecutive patient series, Interi effectively evacuated and removed blood and fluid from the internal surgical spaces in patients undergoing lipo-abdominoplasty documented on HRUS. Through an average 5.3-month follow-up period, 3/71 (4.2%) patients developed seromas. Although this study had no specific control group, our prior seroma rate with standard drains in immediate 100 patients’ pre-Interi was 22%. In addition, there are thousands of clinical studies reporting seromas with historical postoperative rates averaging 10% to 20% or greater with standard drains; (2) ICS's ability to approximate and hold together surgical tissue planes in abdominoplasty was easily visualized on HRUS in all imaged patients confirming ultrasound should be considered the new standard of care in evaluation of fluid collections; (3) Documented adverse events and complications including seroma, infection, flap loss, and all other complications in our study were equivalent or lower than complications in historical studies utilizing the current standard of care with surgical drains; (4) This is the first documentation of internal tissue closure adiographically and identifies HRUS as a new method to follow patients and study fluid collection clinically and in research.

ICS's greatest impact clinically thus far has been in larger body contouring aesthetic and concologic procedures such as breast reconstruction; however, every surgical subspecialty will likely be impacted. Further prospective comparison studies and additional surgical applications will be required to further define Interi's future effectiveness and indications. We believe higher powered systems such as ICS have significant advantages over the current standard of care and may create a brand new class of medical device particularly for larger critical surgical procedures.

## Supplementary Material

ojac073_Supplementary_Data
